# Fully printed flexible and disposable wireless cyclic voltammetry tag

**DOI:** 10.1038/srep08105

**Published:** 2015-01-29

**Authors:** Younsu Jung, Hyejin Park, Jin-Ah Park, Jinsoo Noh, Yunchang Choi, Minhoon Jung, Kyunghwan Jung, Myungho Pyo, Kevin Chen, Ali Javey, Gyoujin Cho

**Affiliations:** 1 Department of Printed Electronics Engineering, Sunchon National University, Maegok, Sunchon, Jeonnam 540-742 Korea; 2 Research Institute of Printed Electronics, PARU Co. Seomyeon, Sunchon, Jeonnam 540-813 Korea; 3 Electrical Engineering and Computer Sciences, University of California, Berkeley, CA 94720

## Abstract

A disposable cyclic voltammetry (CV) tag is printed on a plastic film by integrating wireless power transmitter, polarized triangle wave generator, electrochemical cell and signage through a scalable gravure printing method. By proximity of 13.56 MHz RF reader, the printed CV tag generates 320 mHz of triangular sweep wave from +500 mV to −500 mV which enable to scan a printed electrochemical cell in the CV tag. By simply dropping any specimen solution on the electrochemical cell in the CV tag, the presence of solutes in the solution can be detected and shown on the signage of the CV tag in five sec. 10 mM of N,N,N′,N′-tetramethyl-p-phenylenediamine (TMPD) was used as a standard solute to prove the working concept of fully printed disposable wireless CV tag. Within five seconds, we can wirelessly diagnose the presence of TMPD in the solution using the CV tag in the proximity of the 13.56 MHz RF reader. This fully printed and wirelessly operated flexible CV tag is the first of its kind and marks the path for the utilization of inexpensive and disposable wireless electrochemical sensor systems for initial diagnose hazardous chemicals and biological molecules to improve public hygiene and health.

Cyclic voltammetry (CV) has been used as a powerful tool for the study of electrochemical redox reactions between the electrodes and the solutions of all kind of solutes such as metals^1^, organic molecules^2^, proteins^3^, bacteria^4^, viruses^5^, and DNA^6^. Because of the high sensitivity of the electron transfer redox reactions, CV could become a very promising ubiquitous electrochemical sensor protocol if the cost and size of the CV system were significantly reduced to commercially viable single-use disposable units for checking traces of hazardous materials such as lead^7^, mercury^8^, arsenic^9^, e-*coli*^10^, and pesticides^11^ in water or to diagnose the level of glucose^12^, cholesterol^13^ and specific enzymes^14^ in blood. This single-use disposable CV would dramatically reduce the cost of maintaining the public health system^15^. Therefore, inexpensive and disposable CV measurement system that can be operated wirelessly using a smartphone or RF (radio frequency) reader without complicate operation processes is in high demand for the realization of an ubiquitous sensor network system (Figure 1a). They would mainly be used as ubiquitous diagnostic and testing tools for detecting and monitoring the level of target specimens. However, there is no technology that is advanced enough yet to build a wireless, inexpensive and disposable CV system. In this paper, as a form of RF-tag, an extremely inexpensive, disposable and fully printed CV system is demonstrated for the first time by mimicking and combining basic CV concepts and wireless power transmission technologies of RF devices. To realize the fully printed CV tag, a key issue of wirelessly generating triangular waveform (±500 mV) that can scan the electrochemical cell at a low frequency (<1 Hz) to set up a redox reaction needs to be addressed through a minimum number of printed thin film transistors (TFTs). Fully printed 13.56 MHz rectenna and a ring oscillator with large trap charges in the channels of printed TFTs were respectively utilized to wirelessly generate triangular waveform by using only 10 printed TFTs.

## Results

**Figure 1:**
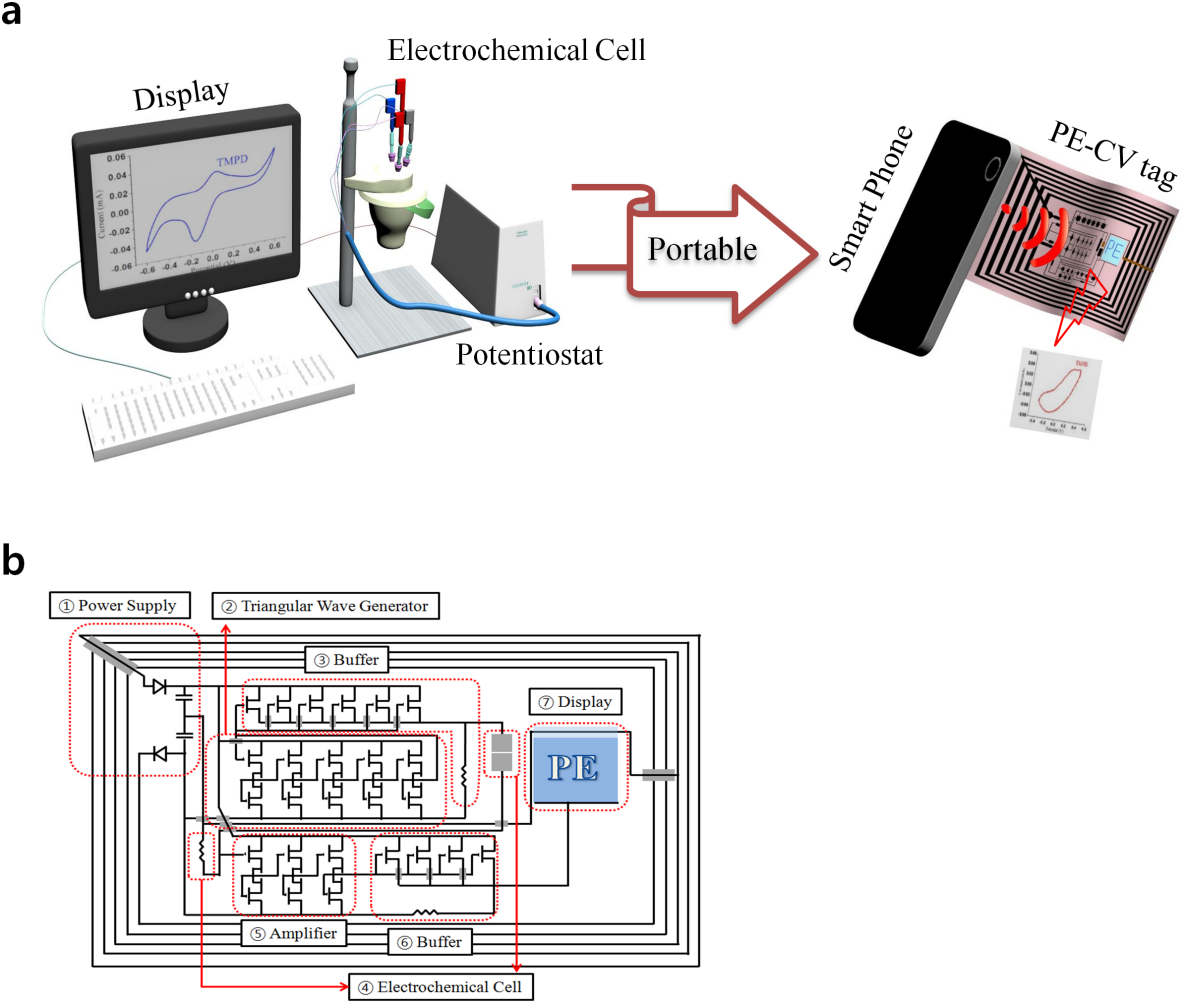
(a) Informative illustration of typical CV system and disposable printed CV tag. (b) Schematic circuit diagram of the gravure printed wireless cyclic voltammetry (CV) tags. The circuit was designed to couple AC power from a 13.56 MHz reader and then convert the coupled AC to polarized DC ±10 V (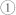 in Figure 1b). Polarized DC will operate the printed ring oscillator to generate a triangular voltage waveform (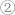 in Figure 1b). The generated waveform will pass through the buffer to meet the impedance difference from the electrochemical cell (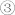 in Figure 1b). The electrochemical cell will run the redox reaction with a single drop of specimen solution (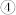 in Figure 1b) by the voltage triangular waveform. The output current of the electrochemical redox reaction will be amplified *via* an amplifier circuit (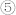 in Figure b) and the signal will pass through the buffer to meet the impedance difference from the printed signage (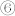 in Figure b). The signage will indicate the concentration level of specimen in the solution (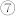 in Figure b). It will indicate whether the concentration is above or below a pre-determined value.

Based on two key units of the wireless power transmitter (Figure 1b-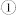) and the triangular wave generator (Figure 1b-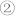), the circuit layout of the fully printed wireless CV tag was designed by using a minimum number of printed thin film transistors (TFTs) to alleviate the issue of V_th_ shift and shown in Figure 1b as a platform of disposable CV system. The circuit of CV tag was fabricated using all scalable printing methods, a roll-to-roll gravure, a roll-to-plate gravure, a drop casting, and a screen printer (Figure S1 in Supplementary Information).

**Figure 2:**
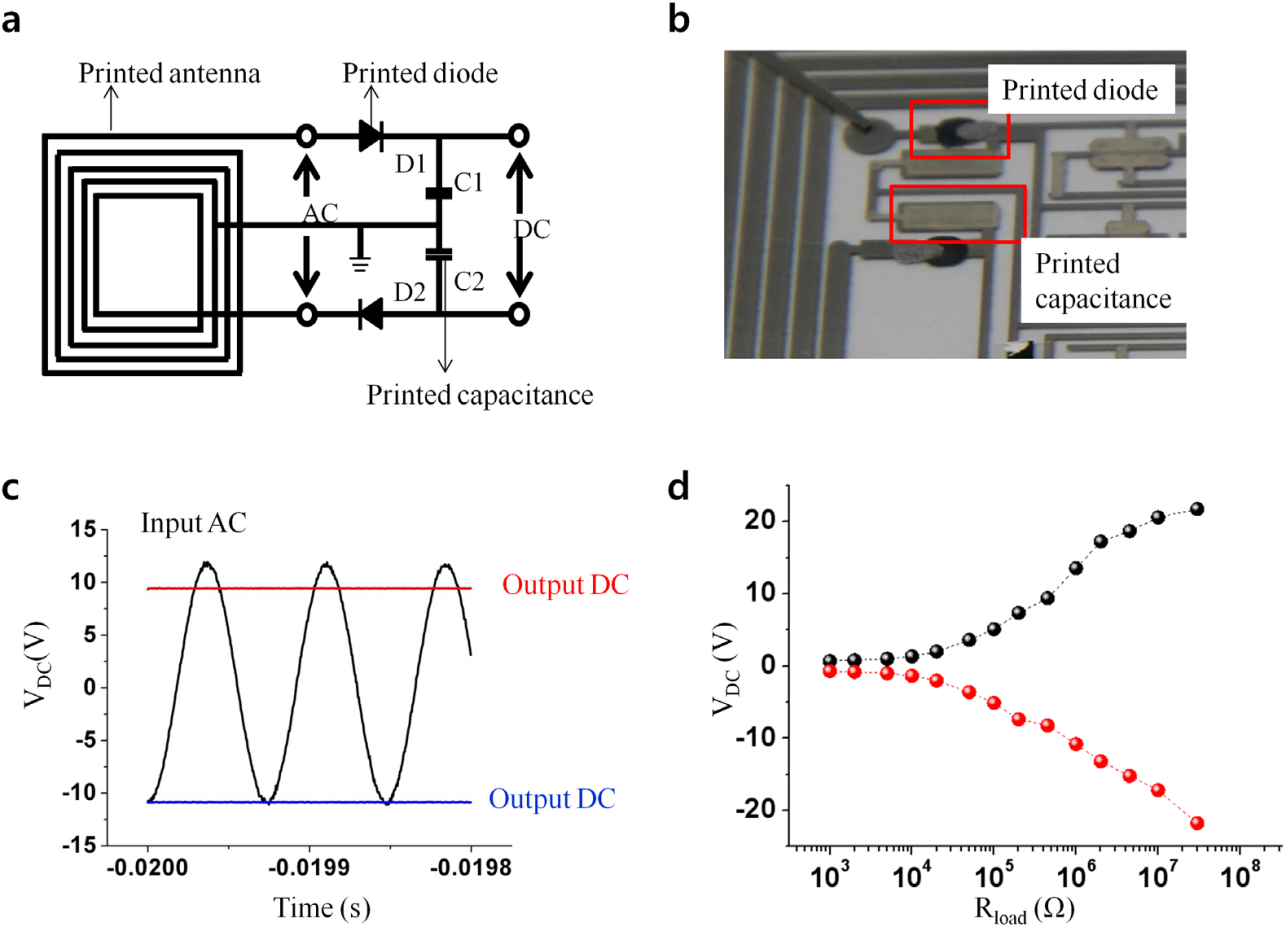
(a) Circuit layout of the printed rectenna to provide polarized DC power, (b) a real image of printed diodes and capacitors, (c) input-output electrical characteristics of the rectifier at 13.56 MHz AC input and (d) output ± DC voltages under variations in load resistance.

To provide a polarized DC voltage from the coupled 13.56 MHz AC signal of the reader, we modified our previously reported R2R gravure printed rectenna^16^,^17^, as shown in the layout in Figure 2a and the resulting printed diodes and capacitors are shown in Figure 2b. The printed diodes showed a rectifying ratio of 10^3^, and capacitances of two printed capacitors were all about 8 nF/cm^2^. The polarized DC voltages (Figure 2c) were measured by placing the printed rectenna on the RF (13.56 MHz) reader with a distance of 2 cm. We adapted a center tap transformer, consisted by divided antenna, 2 diodes and 2 capacitors, to provide the + and − DC voltage for printed triangle wave generator. Using a load of 1 MΩ, voltages of +9.4 V and −10.8 V DC were attained for the optimized printed antenna by monitoring the rectified polarized DC voltages and varying the values of the inductance of the antenna (Figure S2 in Supplementary Information). The polarized DC voltage was also decreased with decreasing load's resistance (Figure 2d).

To generate a cyclic waveform with a low frequency (<1 Hz) from the rectified polarized DC voltage, fully gravure printed *p*-type SWNT-based channel network thin film transistors (*cn*TFTs) were used to construct a five stage ring oscillator with *p*-type inverters. The *cn*TFTs can operate under less than 20 V and their mobility range (0.01 to 10 cm^2^/Vs) can be controlled through the loading concentration of SWNT in the ink^18^,^19^,^20^,^21^. To print the five stage ring oscillator, we used the same silver and BaTiO_3_ inks as in the printing of the rectenna for reducing a number of printing steps while SWNT ink was used for printing the channel of the *cn*TFTs. To manufacture the ring oscillator, roll-to-plate (R2P) gravure was used to print 10 *cn*TFTs with a gate width of 300 μm and channel length of 160 μm for both the drive and load *cn*TFTs (Figure S3 in Supplementary Information). The optimized cell depth of gravure plate was 37–40 μm with the cell wall thickness of 50–55 μm for the printing active layers of drive and load TFTs using low viscosity of SWNT based ink (~30 cp) (Figure S4 in Supplementary Information). By repeating the printing of active layers on load TFTs, a higher SWNT network density can be achieved to generate higher current in the channels while thicknesses of all printed gate, dielectric and drain-source electrodes are kept same (Figure S5 in Supplementary Information).

**Figure 3:**
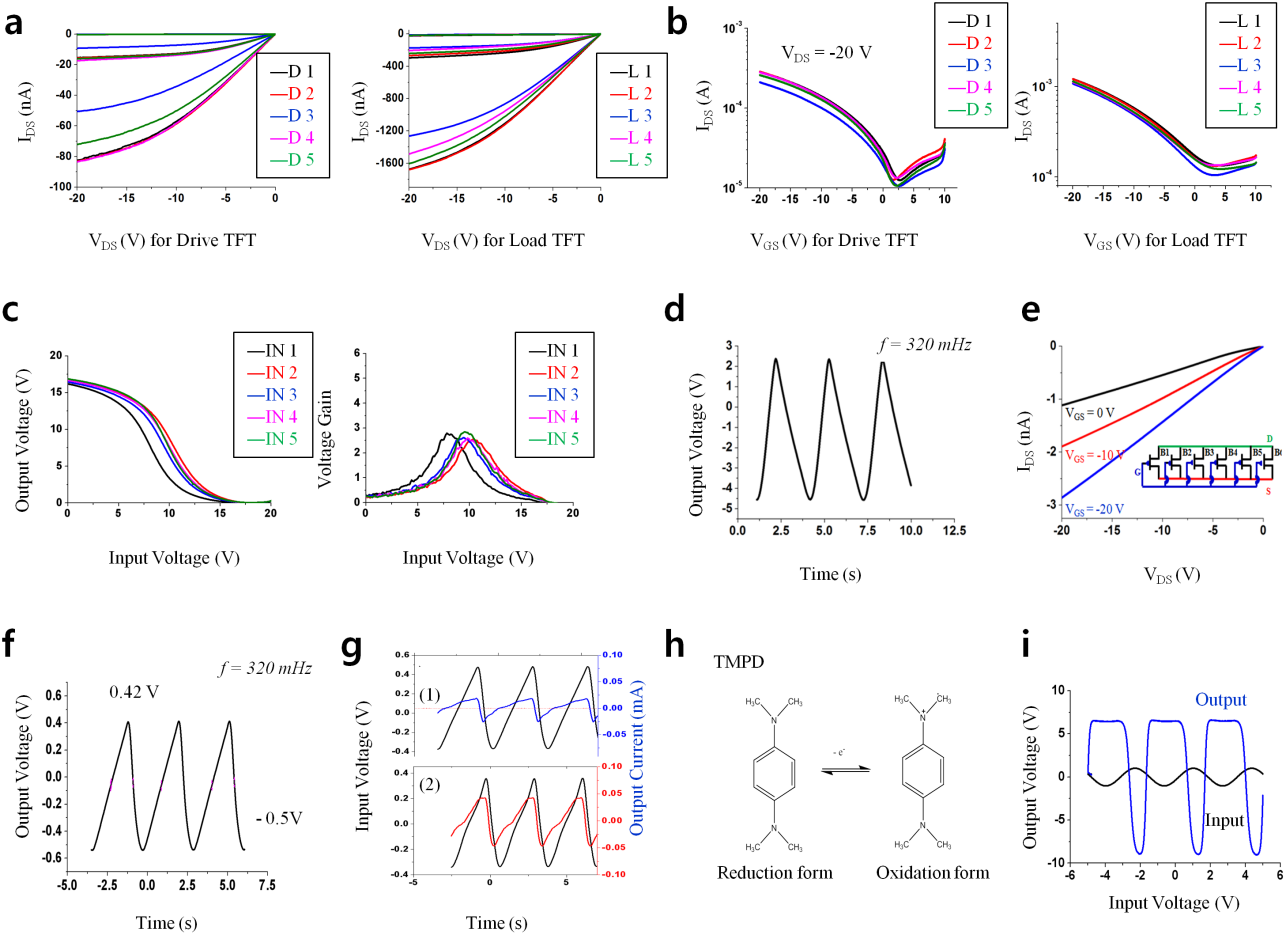
(a and b) Output and transfer characteristics of the printed *cn*TFTs for 5 drive and 5 load TFTs respectively in the printed ring oscillator. (c) Electrical characteristics of inverters and (d) output characteristic of the ring oscillator. (e) Total output characteristics of the printed buffer unit consisting of 6 *cn*TFTs and a resistor (measured based on contacting gate and drain-source electrodes as shown in the inset circuit). (f) Modified triangular wave following the buffer unit. (g) Generated signals before scanning (black) and after scanning the electrochemical cells without (1, blue) and with TMPD (2, red) in a drop of solution. (h) TMPD structure for oxidation and reduction reaction. (i) The input and amplified output signals after passing through three amplifying inverters.

Their output and transfer characteristics are shown in Figure 3a and 3b respectively. Based on the attained transfer characteristics, the mobility (μ), transconductance (g_m_), on-off current ratios from I_DS_-V_DS_ and threshold voltages (V_th_) for both five drive *cn*TFTs and five load *cn*TFTs were extracted and shown in Table S1 (Supplementary Information). The shift range of V_th_ from the fully gravure printed 10 *cn*TFTs were about ±0.4 V for the drive *cn*TFTs and ±0.6 V for load *cn*TFTs. Those shift ranges are narrow enough to run the printed ring oscillator for generating the triangular wave. Each inverter delivered a gain of 3 (Figure 3c), and the ring oscillator with five inverters generated a pseudo triangular waveform with a voltage amplitude of 7 V at 320 mHz (Figure 3d). In fact, the pseudo triangular wave can be only generated by the printed *cn*TFTs based ring oscillator because of the parasitic and trapped channel capacitances due to carbon nanotube network structures in *cn*TFTs. Although we only showed the frequency of 320 mHz at here, the frequency can be varied from 0.3 Hz to 2 Hz based on the printed network density of SWNT of printed *cn*TFTs. This is an advantage of our printed *cn*TFTs for scanning electrochemical cell in CV tag over other technologies such as amorphous silicon TFTs where only a sine wave with a designed single frequency can be generated because of no trap capacitance in the channel (Figure S6 in Supplementary Information). The frequency of the triangular waveform can be fine-tuned in the range of 0.3 Hz to 2 Hz as shown in the Figure S7 (Supplementary Information) by simply changing loading concentration of SWNTs.

When the generated triangular waveform is supplied directly into the printed electro-chemical cell (Figure S8 in Supplementary Information), wherein printed silver and carbon electrodes were used with a poly(ethylene oxide) and LiCF_3_SO_3_ gel type electrolyte, the output voltage range is reduced to zero because of improper impedance matching between the electrochemical cell (10 KΩ) and ring oscillator (1 MΩ). Therefore, a buffer unit is needed to match the impedance levels between the ring oscillator and electrochemical cell. The printed buffer unit (Figure 1b-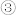) with high on currents and low on-off current ratios (Figure 3e) is consists of 6 *cn*TFTs and a resistor (~7 KΩ). In the printed CV tag, three resistors were printed by screen printer using a carbon paste (DC-20, purchased from Dozen Co. Korea) for the buffer units (Figure 1b-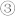 and 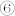) and the electrochemical cell (Figure 1b-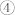). Their electrical parameters are listed in Table S1 (Supplementary Information). The 6 *cn*TFTs of buffer unit were printed using R2P gravure with high SWNT concentration in the semiconducting ink. Each *cn*TFTs exhibited on-currents in the range of 400 ~ 500 μA with a very low on-off ratio due to the enhanced metallic percolation in the dense SWNT networks. The buffer units with low on-off ratio were employed to reduce the input voltage to ±500 mV (Figure 3f) which is the appropriate scanning range of the electrochemical cell. The printed electrochemical cell with two electrodes was proven to be well operated under commercial CV (Figure S9 in Supplementary Information). The resulting current after scanning printed electrochemical cell in the CV tag can be converted to voltage by connecting the printed resistor (Figure 1b-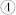: ~5 × 10^3^ ohm) which provides the output voltage for electrochromic indicator and re-plotted into the cyclic voltammogram.

Because these output voltage (±70 mV) and current (~40 μA) were low after scanning the electrochemical cell, they need to be amplified to turn on the electrochromic indicator for showing pre-determined concentration (10 mM) level of N,N,N′,N′-tetramethyl-p-phenylenediamine (TMPD, purchased from Aldrich) as a standard reference in this work (Figure 3g). After running the redox reaction of TMPD (Figure 3h) in the printed cell, the output voltage and current needed to be amplified to reach 1.5 V and 500 μA, respectively, to provide sufficient power to display the letters “PE” as an indicator of the presence of TMPD in the specimen. To construct the amplifier, *cn*TFTs based three inverters were printed with a gain of three to five (Figure S10 in Supplementary Information) to amplify the output signal as shown in Figure 3i. The electrical characteristics of the 6 *cn*TFTs in the inverters with extracted mobility, transconductance, on-off current ratios and threshold voltage are shown in Table S2 (Supplementary Information). As the impedance was matched between the ring oscillator and the electrochemical cell using the buffer unit, another buffer unit (Figure 1b-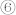) is also needed between the electrochromic signage (20 KΩ) and the amplifier (1 MΩ) for impedance matching. The electrical parameters of the buffer are listed in Table S2 (Supplementary Information).

The amplified output signal was used to run the reduction and oxidation of the patterned conducting polymer in the electrochromic signage, and thus the blinking signage will be used to indicate the presence of chemicals in the specimens. The signage was fully printed and attached on printed CV tag. A clear concept of the printing sequence for the electrochromic signage was given in Figure S11 in Supplementary Information.

## Discussion

**Figure 4:**
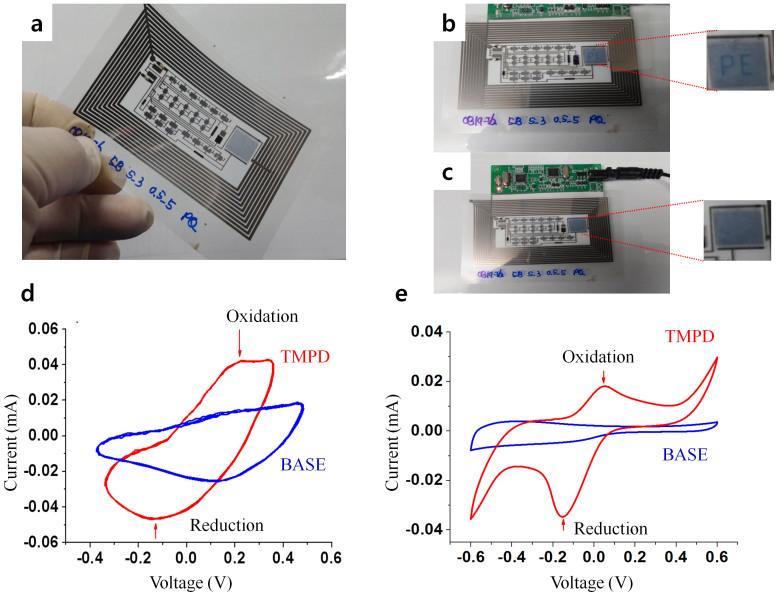
(a) Optical image of fully printed wireless cyclic voltammetry tag. (b) Operation images of CV tag with and (c) without TMPD in the solution on 13.56 MHz reader. (d) Converted cyclic voltammogram from the printed wireless CV tag *vs* (e) a commercial CV instrument (please refer the interconnected video file for the demonstration of wireless CV tag operation in Figure S12 in Supplementary Information).

The fully printed flexible CV tag was completed by assembling the printed circuits including ring oscillator, buffer, electrochemical cell, amplifier, and signage onto the previously R2R gravure printed rectenna. The resultant CV tag is shown in Figure 4a. The working concept of the wireless and flexible CV tag is demonstrated in the following sequences (watch the video file by clicking Figure S12 in Supplementary Information). After dropping 500 μl of TMPD solution (10 mM) on the printed electrochemical cell, the CV tag was placed on the custom made RF (13.56 MHz) reader (Figure S13 in Supplementary Information). The antenna was subsequently coupled to 13.56 MHz AC. The coupled AC was rectified into polarized DC (> ± 10 V) through two diodes and two capacitors which caused the ring oscillator to generate a pseudo triangular waveform with output voltage of 7 V at 320 mHz. This was then passed through the buffer unit to scan the electrochemical cell. The output signal after scanning the electrochemical cell was amplified to turn on the signage according to the concentration level of TMPD in the solution. In this work, whenever the concentration of TMPD was higher than 10 mM, the signage “PE” blinked (Figure 4b) while it did not show anything (Figure 4c) when it was lower than 10 mM. Furthermore, clear cyclic voltammograms for scanning the electrochemical cell with and without 10 mM of TMPD were obtained by re-plotting the output triangular voltage waveform (Figure 4d). The attained half potential (E_1/2_ ~ 0.05 V, oxidation potential is 0.22 V and reduction potential is −0.17 V) of TMPD from the CV tag was nearly identical to the value obtained from a commercial CV instrument, SP-240, Biologic, which uses the same frequency for the triangular voltage waveform and printed electrochemical cell (Figure 4e). However, when the generated triangle wave frequency is higher than 0.6 Hz in the CV tag, the clear redox peaks cannot be observed as shown in Figure S14 (Supplementary Information). Furthermore, as a typical example of testing a specimen in aqueous solution, 10 mM of K_3_(FeCN)_6_ aqueous solution was checked using the CV tag, and results of converted cyclic voltammogram was shown in Figure S15 (Supplementary Information). Those results support that the CV tag can examine specimens not only in organic solution, but also in aqueous one.

Up to present, there has been no commercial success in fully printed TFT-based electronic devices due to some kind of difficulties in integrating a number of printed TFTs through a scalable printing method. As increasing a number of integrated TFTs, the range of threshold voltage shits of TFTs needs to be controlled as narrow as possible to properly operate designed function of printed devices. However, the scalable printing method is not fully understood to integrate a number of TFTs on plastic foils while keeping the constant range of threshold voltage shifts because of difficulty in controlling a constant charge transport through the printed channels. Therefore, the printed TFT-based devices should be constructed by using a minimum number of printed TFTs while a function of printed device needs to be competitive over Si based one. Since the fully printed CV tag required the integration of only 26 *cn*TFTs, the acceptable range of threshold voltage shits of *cn*TFTs is controllable and achievable by using a scalable printing method such as a roll-to-roll gravure. Furthermore, the unique function of printed CV tags cannot be simply reproduced using a Si technology with a comparable cost to the R2R printing process. Therefore, the fully printed CV tag will be the first leading model to show a way of how printed TFT-based electronic devices should go for the successful launching in the IT markets.

In the core of the printed CV tag, fully gravure-printed *cn*TFTs were employed for constructing a circuit consisting of a ring oscillator to generate triangular waveforms, buffer units for an impedance matching, and an amplifier for enhancing output signal to turn on the signage. Those simple units can be combined with a variety of printable sensors and RF devices to create novel devices with an extremely low cost. In other words, the printed rectenna, ring oscillator, buffer and amplifier units in the printed CV tag will be key elements for the construction of variety of printed RF-sensors, this CV tag will be used as a platform for the further construction of a variety of RF based electrochemical sensors.

In conclusion, we have presented a fully printed flexible CV tag with the size of 9.5 × 5.5 cm^2^ that can operate wirelessly using a 13.56 MHz RF reader. However, for the practical purpose, the size of the CV tag can be reduced up to the half (4 × 2 cm^2^) of the current one by optimizing spaces between *cn*TFTs in the circuit of the CV tag and reducing the operation power to less than DC 5 V as the current size of 13.56 MHz antenna is designed to provide maximum coupled AC power using a sufficient length of printed antenna pattern (higher inductance). Furthermore, although we utilized R2P gravure, screen printer and drop casting method for the convenience of fabricating the CV tag in the Lab, the whole fabricating process can be repeated by R2R gravure because the rectenna, ring oscillator, buffers, electrochemical cell, amplifier and signage in the CV tag were all designed to compromise the limit (±20 μm) of a registration accuracy of a current R2R gravure system. Therefore, this technology could be used as a platform for disposable wireless electrochemical sensors to a variety of specimens in aqueous and organic solutions to monitor or diagnose pathogens and hazardous materials by modifying the electrodes of the electrochemical cells.

## Methods

### Roll-to-roll (R2R) gravure printed antenna, bottom electrodes, gate electrodes and wires

Two color units of R2R gravure system (Taejin Co. Korea: Figure S1 in the Supplementary Information) was employed to print antenna, bottom electrodes and wires on a roll of poly(ethylene terephthalate) (PET) film (width of 200 mm and thickness of 100 μm, purchased from SKC, Korea) with silver nanoparticle based conducting ink (PG-007 BB type, Paru Co. Korea) and the web transfer speed of 8 m/min under roll pressure of 0.5 MPa and web tension of 60 N. The silver ink was further formulated to meet the viscosity of 500 cp and surface tension of 47 mN/m using respectively ethylene glycol (Aldrich) and Dipropylene glycol methyl ether (Aldrich). The curing time of printed silver layer was 10 sec by passing through a heating chamber of 150°C, installed in R2R gravure system. The resulting printed silver patterns on PET roll showed the thickness of 450 (±50) nm without any defects and were shown in Figure S16a (Supplementary Information).

### Roll-to-plate (R2P) gravure printed ring oscillator, buffer, and amplifier

Full R2P gravure system (Figure S1 in the Supplementary Information) was employed to print single walled carbon nanotube network based thin film transistors (*cn*TFTs), used as a building block to construct all the integrated circuit of printed cyclic voltammetry (CV) tag (Figure 1). 26 *cn*TFTs for the integration of circuit in printed CV tag were printed on the previously R2R gravure printed film (printed antenna, bottom electrodes and wires with thickness of 450 nm; Figure S16b in Supplementary Information) following printing sequences. First, BaTiO_3_ nanoparticle based dielectric ink (PD-100, Paru Co., Korea) was further formulated to meet viscosity (75 cP) and surface tension (31.8 mN/m) using the ethyl 2-cynoacrylate (Aldrich). As shown in R2P gravure printing scheme (Figure S17 in Supplementary Information), the homogeneous and defect free dielectric layers were first R2P gravure printed with a printing speed of 200 mm/s and roll pressure 7.5 kg_f_ on previously printed gate electrodes and bottom electrodes (for capacitors) with a thickness of 2.3 (±0.1) μm and width of 940 (±2) μm (Figure S5 in Supplementary Information). The printed dielectric layers were cured for 1 min 20 sec at 150°C. Second, single walled carbon nanotube (SWNT) based semiconducting ink (PR-040, Paru Co., Korea) was diluted by diethylene glycol monobutyl ether (Daejung Co., Korea) to 1:1 volume ratio for printing active layers using R2P gravure on the printed dielectric layers with a printing speed of 350 mm/s and a roll pressure of 4 kg_f_. The viscosity and surface tension of diluted SWNT ink were respectively 30 cP and 29 mN/m. The resulting SWNT printed films were dried 1 min at 150°C. For using *cn*TFTs in the buffer unit, we used the dilution volume ratio of 5:1 between SWNT ink (PR-040, Paru Co., Korea) and diethylene glycol monobutyl ether for printing only on the TFTs in the buffer unit to increase SWNT network density for providing the high current (Figure S5 in Supplementary Information). Finally, drain-source electrodes were R2P gravure printed using silver nanoparticle based ink (PG-007 AA type, Paru Co., Korea) on SWNT printed film with a viscosity of 800 cP and a surface tension of 42 mN/m. The R2P gravure printing was carried out under a printing speed of 200 mm/s and a role pressure of 5 kg_f_.

### Diode fabrication

Although Shottky diodes were able to print using R2R gravure based on our previous reported process^16^,^17^, the drop casting method was used for the convenience in this work. First, dropping ZnO based hybrid semiconducting ink (PD-070, Paru Co., Korea) on printed silver electrode and then, Al ink (PA-009, Paru Co., Korea) was dropped on the top of ZnO ink. The resulting diodes were further cured for 5 min at 120°C.

### Measurements

Formulated and diluted inks were characterized using DCAT21 (Dataphysics Co., Germany) and SV-10 Vibro viscometer (AND Co., Japan) for respectively measure surface tension and viscosity. All measurements in this work were carried out under ambient condition if there are no other comments. Resistance of printed antenna, bottom electrodes, gate electrodes and wires were measured using two probe methods with HP3457A multimeter. Both surface morphology and thickness of printed layers was characterized using surface profiler (NV-2200, Nanosystem, Korea). LCR meter (E4980A, Agilent, USA), semiconductor parameter analyzer (4155C, Agilent, USA) and digital phosphor oscilloscope (DPO 4054, Tektronix, USA) were respectively used to characterize printed *cn*TFT based circuits.

## Author Contributions

Y.J., A.J. and G.C. designed the experiments. Y.J., H.P., J.P., Y.C. and M.J. carried out the experiments. J.N., K.J. and K.C. performed device simulations. Y.J. and K.C. performed mobility calculations. Y.J. and M.P. performed electrochemistry and analyzed electrochemical data. Y.J., H.P., M.P., A.J. and G.C. contributed to analyzing the data. M.J., M.P., A.J. and G.C. wrote the paper while all authors provided feedback.

## Supplementary Material

Supplementary Information Demo movie of printed CV tag

Supplementary Information Supplementary Information
