# Supplementary material for: Fully printed flexible and disposable wireless cyclic voltammetry tag
Source: Sci Rep. 2015 Jan 29;5:8105. doi: 10.1038/srep08105 (PMC4309959; doi:10.1038/srep08105)
Supplement: Supplementary Information [file srep08105-s2.pdf]

Fully printed flexible and disposable wireless cyclic voltammetry tag

Younsu Jung[1], Hyejin Park[1], Jin-Ah Park[1], Jinsoo Noh[1], Yunchang Choi[1], Minhoon Jung[2], Kyunghwan Jung[2], Myungho Pyo[1], Kevin Chen[3], Ali Javey[3] and Gyoujin Cho[1*]

[1]*Department of Printed Electronics Engineering, Sunchon National University, Maegok, Sunchon, Jeonnam 540-742 Korea*

[2]*Research Institute of Printed Electronics, PARU Co. Seomyeon, Sunchon, Jeonnam 540-813 Korea.*

[3]*Electrical Engineering and Computer Sciences, University of California, Berkeley, CA 94720*

Supplementary Information

Figure S1.

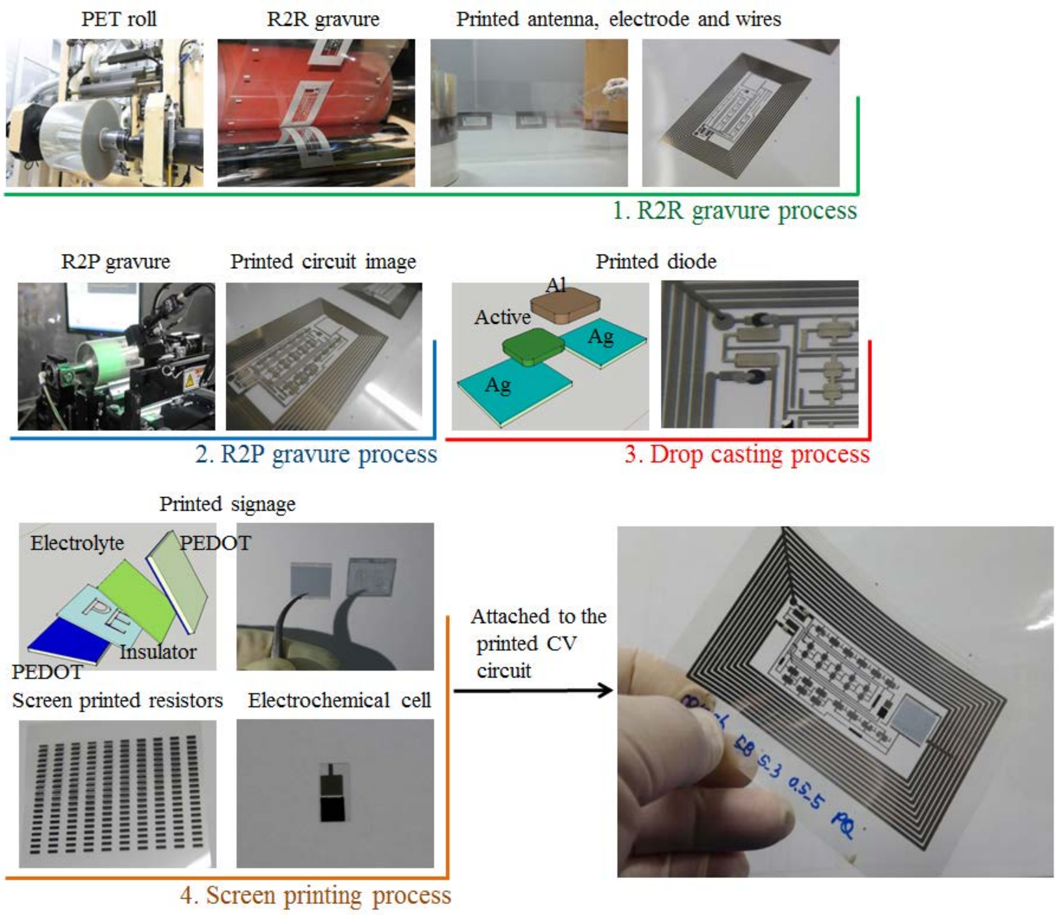

Step by step description of printing process for fabricating flexible wireless cyclic voltammetry tag.

Figure S2.

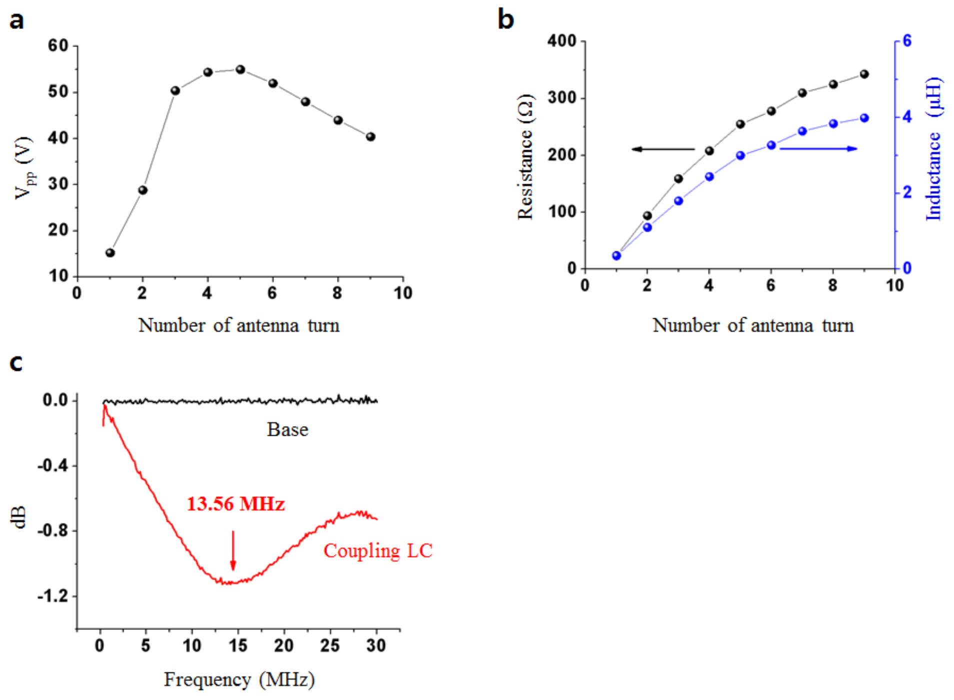

(a) Coupled AC from 13.56 MHz input frequency and (b) measured inductances and end-to-end resistances based on the antenna coil turns, (c) antenna with 5 coil turns provides the maximum resonator quality factor at 13.56 MHz.

Keeping the constant channel length of printed drain-source electrodes is a key control factor for attaining small variation of threshold voltages from printed *cn*TFTs. The fully printed *cn*TFTs showed almost constant channel length and morphology of printed drain-source electrodes as shown in Supplementary Figure S3.

Figure S3.

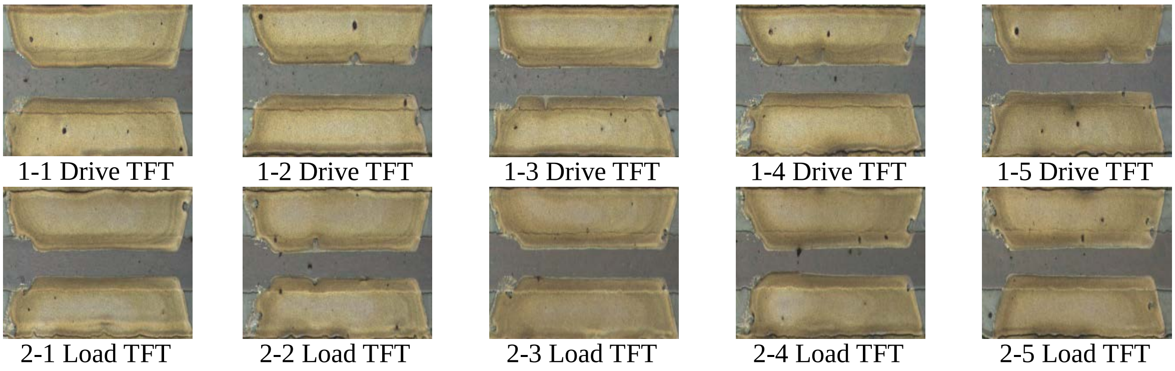

Optical images of drain-source electrodes from 10 of fully printed *cn*TFTs (5 drive and 5 load TFTs) of ring oscillator to generate triangular wave with less than 1 Hz.

Figure S4.

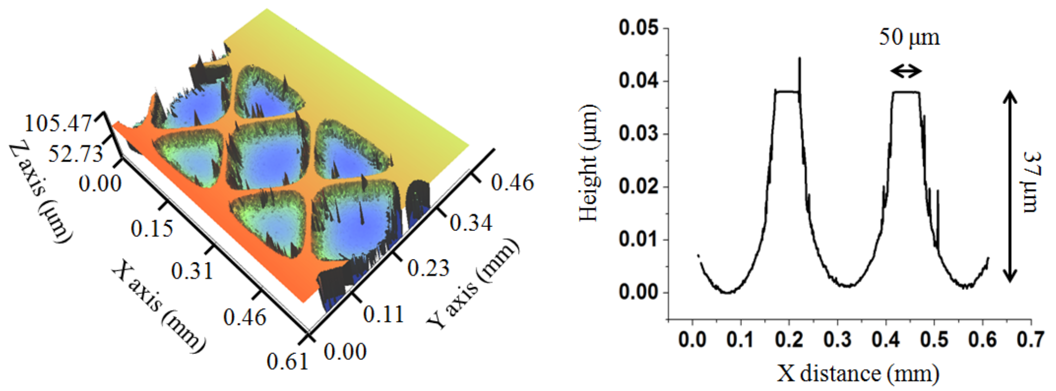

Image of engraved gravure plate for printing active layers with 37 µm depth

Cross sectional SEM images (Supplementary Figure S5a) for printed *cn*TFTs of ring oscillator, buffer, amplifier show almost same thickness of printed gate electrodes, dielectric layers and drain-source electrodes. However, the network density of printed SWNT at each channels from ring oscillator, buffer, amplifier are all different as shown in Supplementary Figure S5b. As increasing the printed network density of SWNTs, the current level is going to raise a couple of mA while the on-off current ratio is dramatically decreasing because of increasing metallic percolation of SWNTs.

Figure S5.

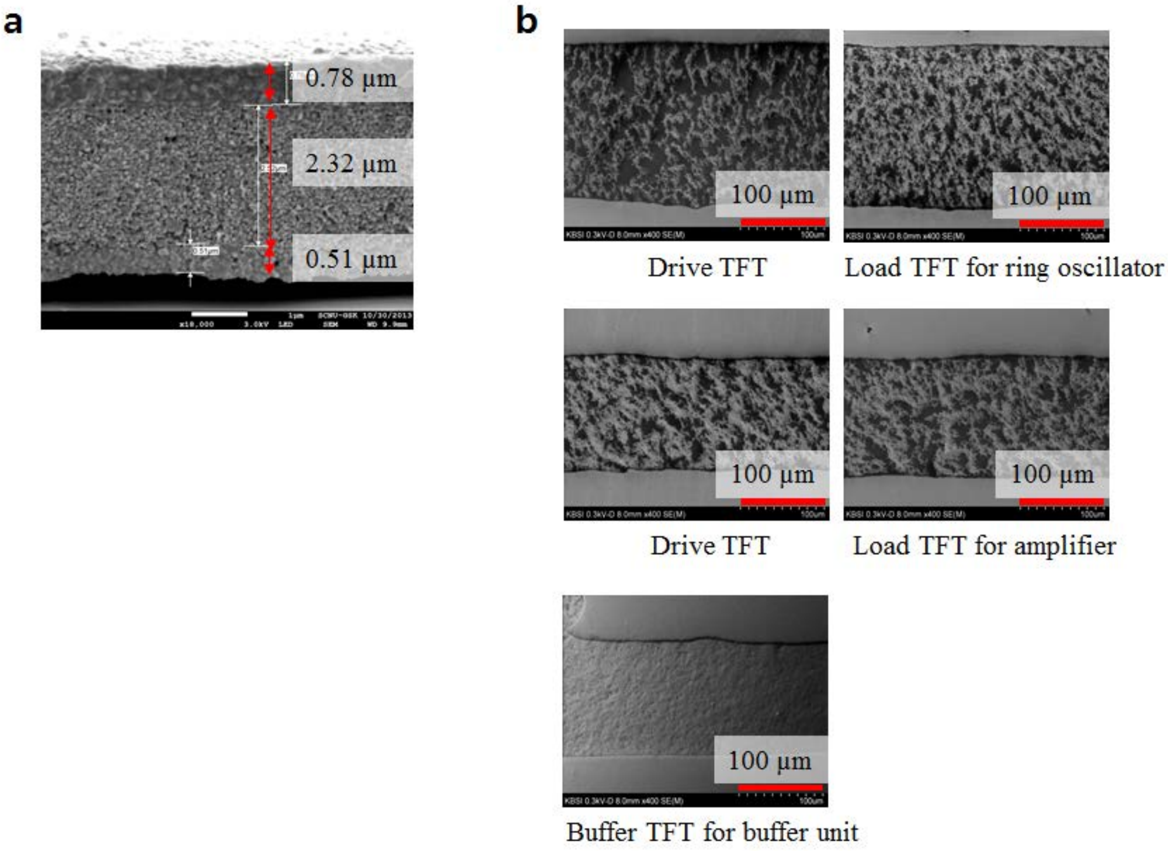

(a) Cross-sectional SEM images for fully printed *cn*TFTs. (b) SEM images for fully printed SWNT networks respectively from ring oscillator, amplifier and buffer.

Figure S6.

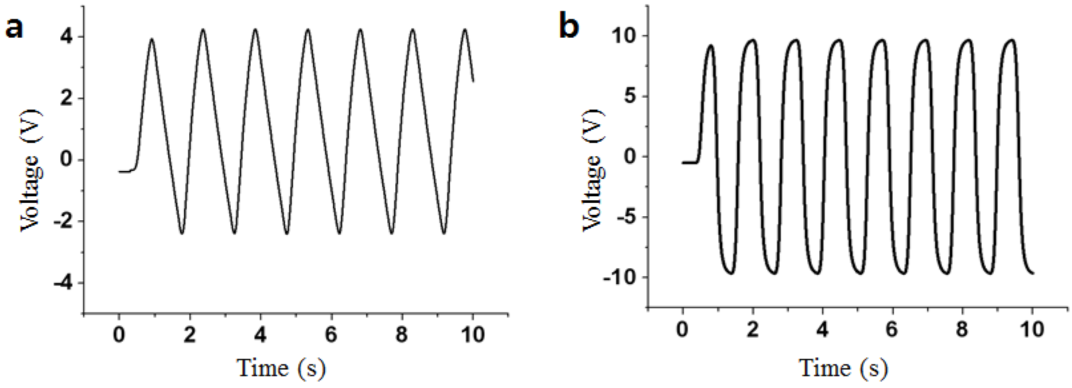

(a) Outputs from a simulation of ring oscillators using the extracted parameters of the *cn*TFTs and (b) amorphous Si based TFTs.

Figure S7.

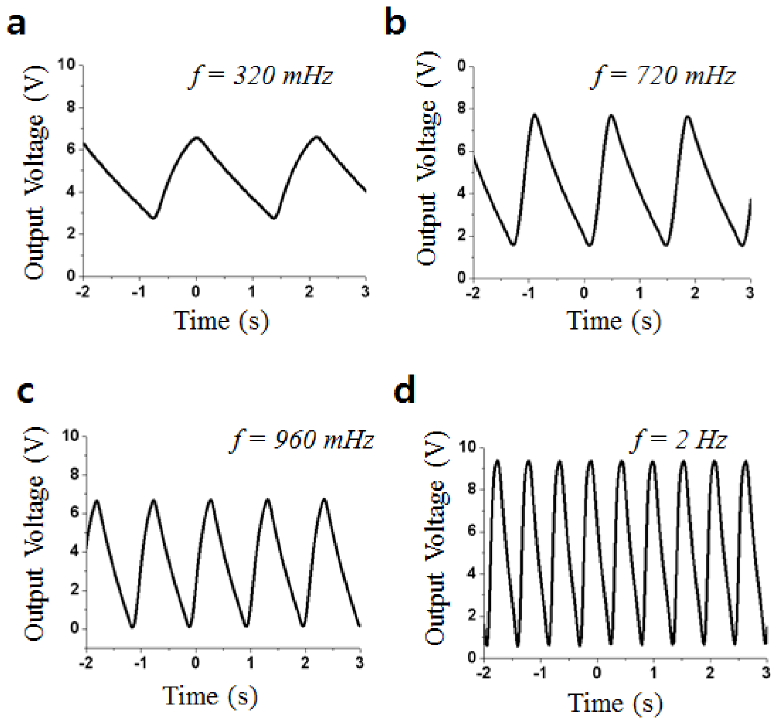

Tailored triangular waveforms with various frequencies (0.3 (A) to 2 Hz (D)) using printed *cn*TFTs based ring oscillators

Screen printer with 250 mesh screen was used to print the working electrode using a carbon paste while the counter electrode was printed using Ag pastes. After printing the two electrodes with a gap of 500 μm, PEO-based $LiCF_3SO_3$ (0.1 M) electrolyte gel with a viscosity of 15,000 cp was screen printed between them with the size of 4000 x 4000 μm^2, as shown in Supplementary Figure S8. The resulting cell was dried under ambient condition and attached to the printed cyclic voltammetry tag using silver glue.

Figure S8.

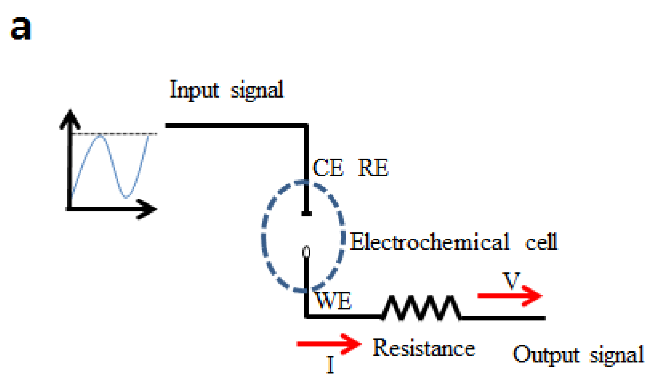

(a) Layout of the printed electrochemical cell and (b) image of printed electrochemical cell. (WE : Working Electrode, CE : Counter Electrode, RE : Reference Electrode)

By employing commercial cyclic voltammetry (SP-240, Biologic), the printed electrochemical cell was tested by dropping one drop of the acetonitrile solution of N,N,N′,N′-tetramethyl-p-phenylenediamine (TMPD) (0 mM, 5 mM and 10 mM) by inputting 420 mHz of triangle wave. The resulting cyclic voltammogram was shown in Supplementary Figure S9.

Figure S9.

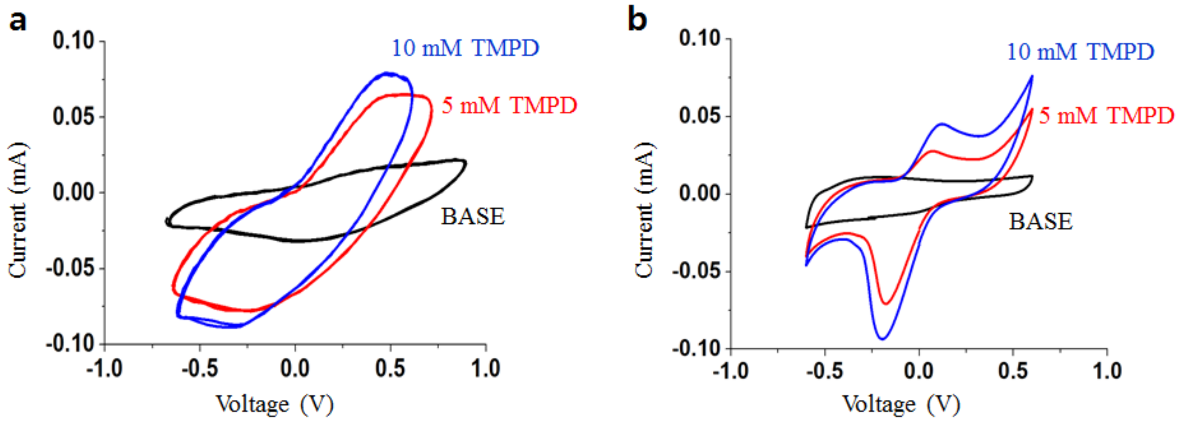

The attained cyclic voltammograms with printed CV tag (a) and commercial potentiostat (SP-240, Biologic) (b) by using printed electrochemical cell with 0 mM, 5 mM and 10 mM of TMPD solution respectively.

Figure S10.

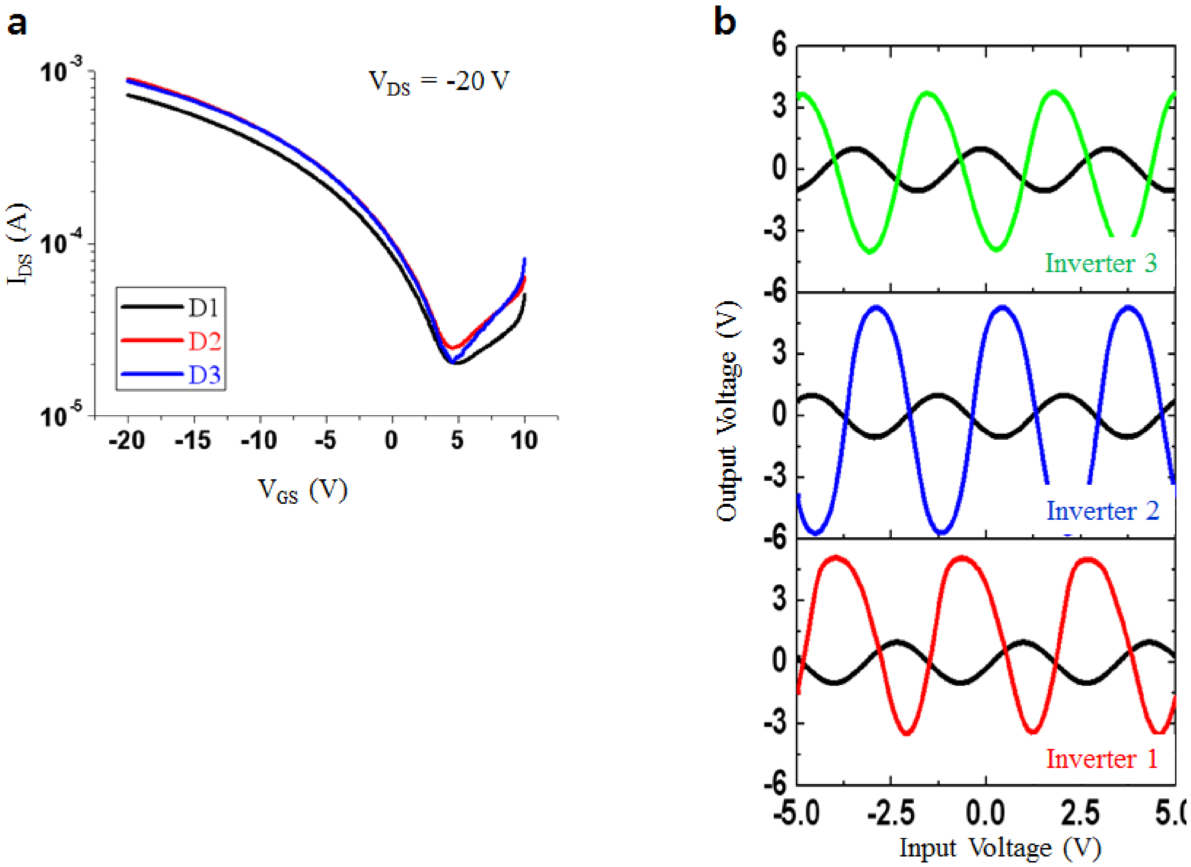

Transfer characteristics (a) of drive *cn*TFTs as well as (b) inverter output by inputting a 2 V AC signal

Layout of printed signage based on conducting polymer PEDOT:PSS. ; The printed signage in this work is constructed by an electrochromic display which can be easily roll-to-roll manufactured. The optimized both surface resistance of poly(ethylene dioxythiophene) doped by poly(styrenesulfonic acid) (PEDOT:PSS) layer and ionic resistance of electrolyte layer were adopted from our previous results to operate the printed signage by less than 3 mW of power. For the fabrication of signage (Supplementary Figure S11), PEDOT:PSS was coated first by R2R coating on PET film with the surface resistance of 20 kΩ/sq and then, insulating pattern was printed on PEDOT:PSS coated film using a screen printer and using poly(methyl methacrylate) (PMMA) as insulating ink. The patterned film by PMMA was dried under 120 °C oven for 10 min and then, further printed by screen printer using electrolyte gel, consisted by poly(ethylene oxide) (PEO) and $LiClO_4$. The resulting film was gently dried under ambient condition for 20 min and then, laminated by another PEDOT:PSS coated PET film to complete the signage. Finally, the attained signage was attached on fully printed CV tag using silver glue.

Figure S11.

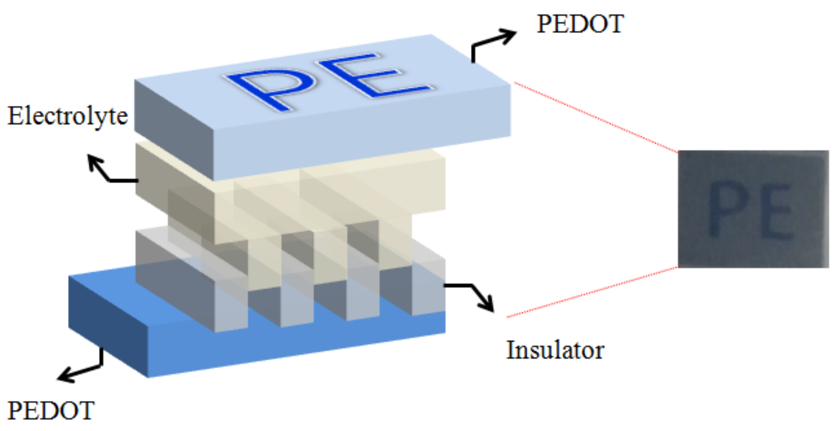

Figure S12.

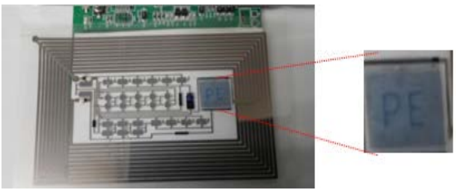

Click above image to watch the video to demonstrate the working process of printed wireless CV tag.

The RF reader was specially manufactured to just provide 13.56 MHz AC following the standard rule of 13.56 MHz RF reader (3Alogics RSK 100) (Supplementary Figure S13). Since this RF reader can be simply replaced by NFC function of smartphone, this disposable wireless CV tag will become ubiquitous sensor in IoT.

Figure S13.

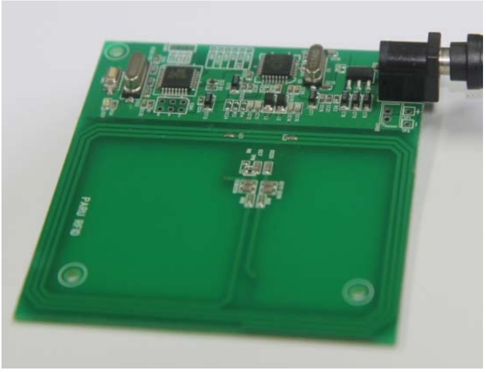

Real image of custom made RF reader powered by 9 V DC.

The printed flexible wireless CV tag with higher frequency of scanning triangle wave than optimized 320 mHz was tested to see whether the electrochemical oxidation and reduction would occur at 10 mM of TMPD solution.

Figure S14.

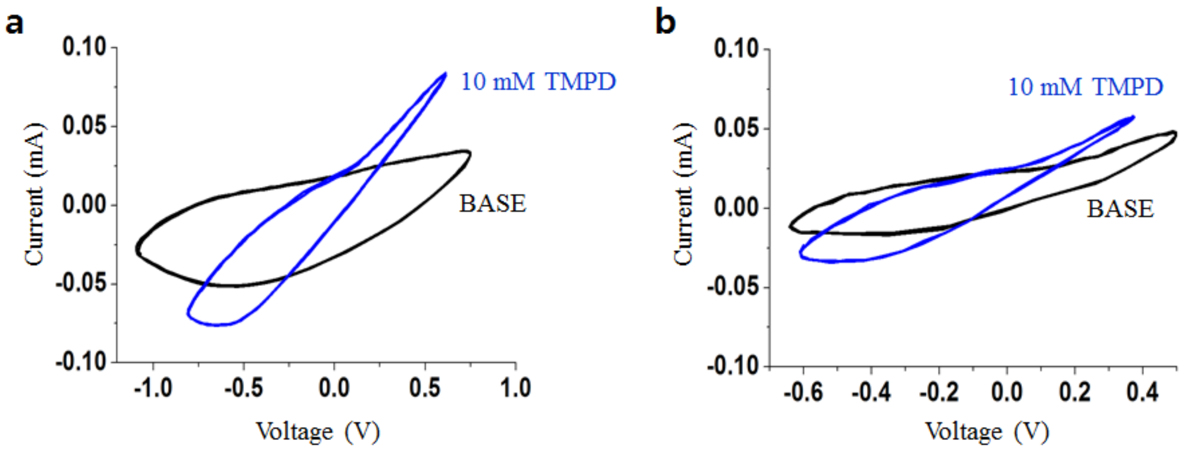

Attained cyclic voltammogram of 10 mM of TMPD by scanning electrochemical cell with the triangle wave frequency of (a) 0.6 Hz and (b) 2.5 Hz in CV tag.

Figure S15.

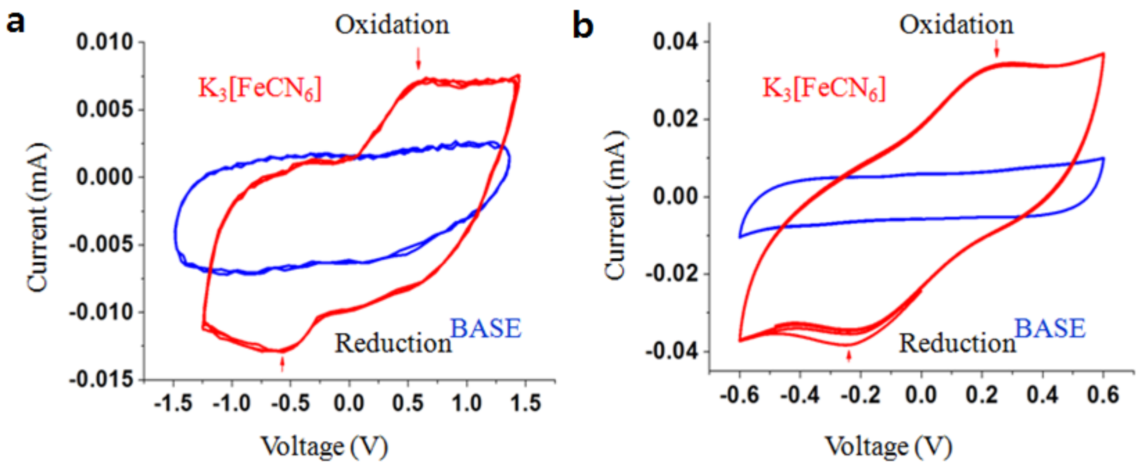

Converted cyclic voltammogram from (a) the printed wireless CV tag with 440 mHz of printed ring oscillator *vs* (b) a commercial CV instrument respectively using 10 mM of aqueous solution of $K_3(FeCN_6)$.

Figure S16.

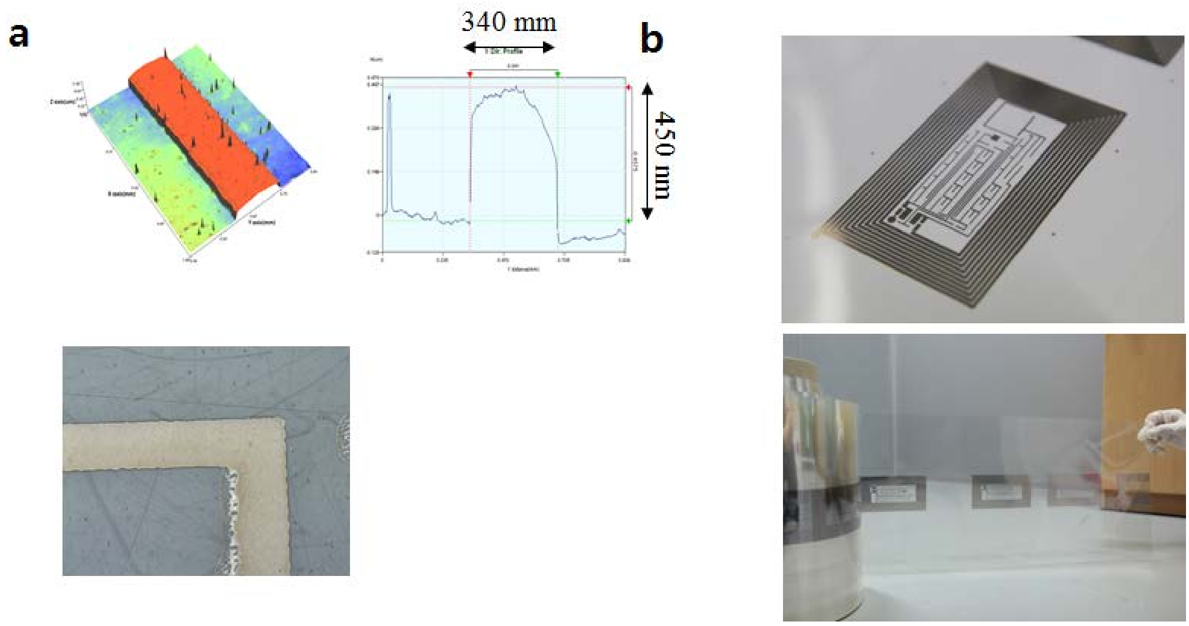

(a) Cross-sectional analysis of printed silver patterns and (b) image of R2R gravure printed antenna, bottom electrodes and wires on PET roll.

Figure S17.

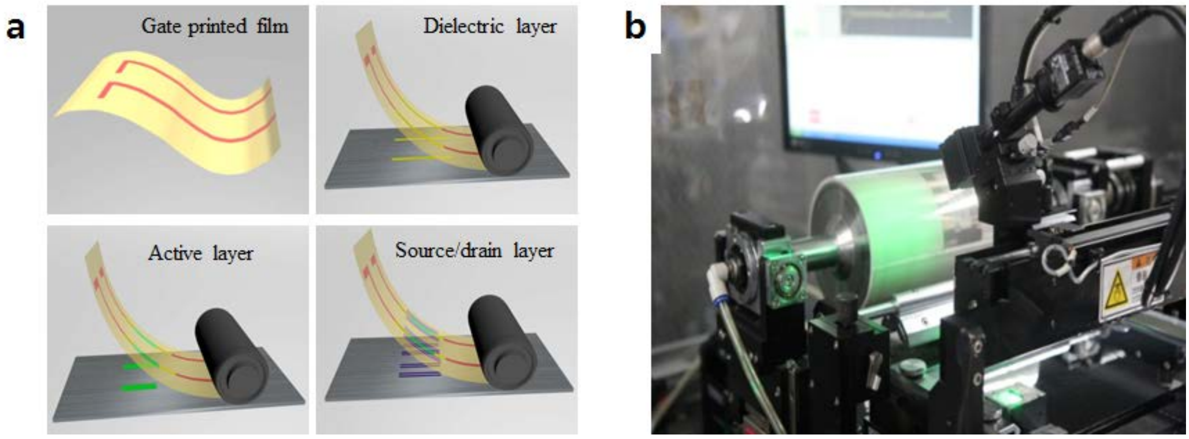

(a) Schematic step for fully R2P gravure printed *cn*TFTs on PET film and (b) real image of R2P gravure used in this work.

Table S1.

TR no	Cap (nF/cm^2)	Channel L/W (μm)	On current (nA)	Off current (nA)	On/off ratio from I_{DS}-V_{DS}	g_m (μS)	V_{th} (V)	μ $(cm^2/V \cdot s)$
D1	8	158/1705	82	0.45	182	14.3	-0.24	0.0047
D2	8	168/1707	83	0.46	180	15.1	-1.17	0.0056
D3	8	160/1740	50	0.27	185	11.0	-1.01	0.0028
D4	8	168/1702	84	0.41	204	14.4	-0.57	0.0051
D5	8	170/1710	72	0.41	175	13.1	-0.32	0.0043
L1	8	174/1721	1680	24	70	65.7	-1.63	0.109
L2	8	174/1709	1680	22	84	69.5	-2.62	0.123
L3	8	166/1690	1260	15	84	61.5	-2.60	0.093
L4	8	179/1616	1490	21	71	65.2	-2.93	0.118
L5	8	178/1600	1610	19	84	65.4	-2.67	0.119
B1	8	121/3556	$5.1x10^5$	$2.1x10^5$	2.4	400	-	1.36
B2	8	103/3627	$5.7x10^5$	$2.3x10^5$	2.5	453	-	1.46
B3	8	102/3639	$4.9x10^5$	$1.8x10^5$	2.7	445	-	1.39
B4	8	97/3617	$4.2x10^5$	$1.6x10^5$	2.6	394	-	0.46
B5	8	97/3628	$4.0x10^5$	$1.4x10^5$	2.9	405	-	1.10
B6	8	105/3556	$3.9x10^5$	$1.5x10^5$	2.6	389	-	1.12
B_{total}	8	-	$2.9x10^6$	$1.2x10^6$	2.	1030	-	-

Extracted electrical parameters (g_m : transconductance, V_{th} : threshold voltage, μ : mobility, and on-off current ratio) from printed *cn*TFTs for a pseudo triangular wave generator and buffer part (D : drive TFT, L : load TFT and B : buffer TFT)

Table S2

TR no	Cap $\left(nF/cm^2\right)$	Channel L/W (μm)	On current (nA)	Off current (nA)	On/off ratio from I_{DS}-V_{DS}	g_m (μS)	V_{th} (V)	μ (cm^2/Vs)
D1	8	148/3500	503	1.69	298	3.51	0.74	0.013
D2	8	148/3480	724	1.75	413	4.42	0.42	0.021
D3	8	152/3502	586	2.00	293	4.16	1.07	0.019
L1	8	149/3500	7230	258	28	131	-0.05	0.181
L2	8	149/3450	5850	128	46	119	-0.66	0.154
L3	8	146/3480	2860	51	56	82	1.34	0.071
B1	8	131/3432	3.8×10^5	1.4×10^5	2.7	387	-	1.43
B2	8	139/3430	4.2×10^5	2.0×10^5	2.1	349	-	1.23
B3	8	132/3443	4.6×10^5	2.0×10^5	2.3	380	-	1.29
B4	8	112/3380	3.6×10^5	1.4×10^5	2.5	366	-	1.33
B_{total}	8	-	1.7×10^6	6.9×10^5	2.5	743	-	-

Extracted electrical parameters (g_m : transconductance, V_{th} : threshold voltage, μ : mobility, and on-off current ratio) from printed *cn*TFTs for the amplifier and buffer part (D : drive TFT, L : load TFT and B : buffer TFT)
